# Trophic state and potential productivity assessment for Qaroun Lake using spatial techniques

**DOI:** 10.1007/s10661-023-11504-2

**Published:** 2023-07-25

**Authors:** Hagar M. Mohamed, Magdy T. Khalil, Ahmed M. El-Zeiny, Nehad Khalifa, Sameh B. El Kafrawy, Wiam W. M. Emam

**Affiliations:** 1 Marine Sciences Department, National Authority for Remote Sensing and Space Sciences (NARSS), Cairo, Egypt; 2 Department of Zoology, Faculty of Science, Ain Shams University, Cairo, Egypt; 3 Environmental Studies Department, National Authority for Remote Sensing and Space Sciences (NARSS), Cairo, Egypt; 4 National Institute of Oceanography and Fisheries, NIOF, Cairo, Egypt

**Keywords:** Carlson trophic state index, Trophometric index, GIS, Fish yield, Chlorophyll-*a*, Eutrophication

## Abstract

Qaroun Lake is one of the most important Egyptian lakes which, recently, have been exposed to severe degradation in water quality and fish productivity. In this manuscript, Carlson’s trophic state index (CTSI) was used to evaluate the trophic state, while the trophometric index (TMI) was used to assess the potential productivity of Qaroun Lake. The present study is one of the initial attempts to investigate these indices in Qaroun Lake. To achieve this work, an integrated multidisciplinary approach was adopted integrating field investigation, geographic information system, and data analysis. CTSI combines three variables of water quality: chlorophyll-a (CHL-a), total phosphorus (TP), and transparency measured by Secchi disk depth (SDD). The result of overall CTSI showed the hypereutrophic state is represented by 62% and eutrophic state is represented by 38% of the total lake’s area. Moreover, the calculated TMI indicated the average potential productivity value (PP) is 619 t. It can be concluded that the hypereutrophic is the dominant state in Qaroun Lake. The present study recommends the application of TMI model to evaluate and monitor the changes in Qaroun Lake’s potential productivity in response to the changing environmental conditions and other biological pressures (e.g., Isopoda paraside).

## Introduction

Qaroun Lake is a closed saline basin in Egypt. It has a great global significance since it compiled the Rmsar convention and was designated as a natural reserve in 1989 (NCS, 2006). Moreover, the local importance is due to it is being the main source of livelihood for the El Fayoum government. In 2014, the lake’s contribution to the country’s total fish yield from inland lakes was about 12.62% (4518 t) (CAPMAS, 2018) which decreased to 2.10% (832 t) in 2018 (CAPMAS, 2018). Napiórkowska-Krzebietke et al. (2016) recorded Mugil cephalus and Solea spp as dominant fish types, while Tilapia zillii and Engraulis encrasicolus were found in the east and the middle of the Lake. Qaroun Lake drainage system is consisting of twelve drains; the main drains are El-Bats and Al-Wadi (Mehanna, 2020; Zaher & Ibrahim, 2018). It receives a massive amount of wastewater reaching 450 mm^3^/year from agricultural sector, sewage water, and excessive nutrient salts from aquaculture drainage dominating the southern part of the lake (Elsayed et al., 2021). Consequently, the lake’s aquatic environment is highly contaminated by inorganic and organic contaminants (El-Zeiny et al., 2019; El Agawany et al., 2021).

Eutrophic phenomena is arisen in Qaroun Lake from the discharge of drainage water into the enclosed lake’s body. The increased nutrient level will increase the rate of phytoplankton growth. Thus, they exceed standard levels which produces high chlorophyll concentration and prevents the sunlight from entering the bottom of the lake causing the transparency factor to decline. High bacterial populations, followed by high breathing rates, cause the loss of living organisms and the release of organic matters. The organic matter is normally bound to bottom sediments, including different forms of phosphorus. Hypereutrophic has a significant impact on fish productivity in the lake (Alprol et al., 2021).

Carlson trophic state index (CTSI) is developed by Carlson (1977) to classify the lake productivity into different categories; oligotrophic, mesotrophic, eutrophic, and hypereutrophic that aid in management plans (Alprol et al., 2021; Carlson & Havens, 2005). The trophic state index (TSI) formula is based on three parameters, i.e., total phosphorus TP, chlorophyll-a (CHL-a), and water transparency (SDD) that finally form CTSI. Geographic Information System (GIS) helps to generate thematic maps for TSI (CHL), TSI (TP), TSI (SDD), and CTSI. Globally, this index is used to assess the lakes eutrophication by many researchers, i.e., Sruthy et al. (2021), Al-Khafaji and Al-Taee (2022), and Yan et al. (2022), and in Egypt, successful attempts were done by Donia and Hussein (2004), Darwish et al. (2021) and Hasan (2021). Stakeholders could easily monitor and assess the eutrophic situation in the lakes to start executing a suitable plan.

Estimating the real fish yield (fish productivity) for water bodies is consuming time and is highly costly (Onumah et al., 2020). As an alternative way, the potential productivity (PP) can be estimated through the changes in the morphometry, water quality data, and biological parameters. Recognizing the potential productivity of lakes is an essential step for good sustainable management. It depends mainly on the determination of the gap between estimated and real yield (Crul, 1992; Tesfaye & Getahun, 2021 and Mohamed et al., 2022). Among the potential productivity models, this manuscript applied the trophometric index (TMI) which has been adopted to assess PP by Lara et al. (2009) on eight reservoirs in the Mediterranean Lakes with model statistical description *R*^2^ > 0.8; *P* < 0.01. Trophometric index is an indirect method to measure the lake’s potential productivity through the combination of morphometry represented in depth, area, perimeter, and volume, chemical factors represented in electrical conductivity (EC) data and biological factors (CHL-*a*) (Milligan et al., 2020).

The combination between GIS and field data could provide a rapid or a large-scale understanding of lake changes and in developing management strategies for the lake (Papastergiadou et al., 2008; Emam, 2016; El-Zeiny et al., 2019; Elkafrawy et al., 2020). Furthermore, the package of GIS could help to achieve the desired purpose by converting the tabulated data to thematic maps to detect the highly affected areas giving an earlier alarm (Hussian et al., 2019).

The main aims of this approach are to quantify and qualify the eutrophic state in Qaroun Lake through mapping the TSI for (CHL-a), (TP), and (SDD) parameters to produce the CTSI map by using the geostatistical analyses, and the second aim is to apply the trophometric index to Qaroun Lake to evaluate its potential productivity. Both CTSI and TMI are specific indices developed to evaluate and understand the productivity of the lake.

## Material and methods

### Study area

**Fig. 1:**
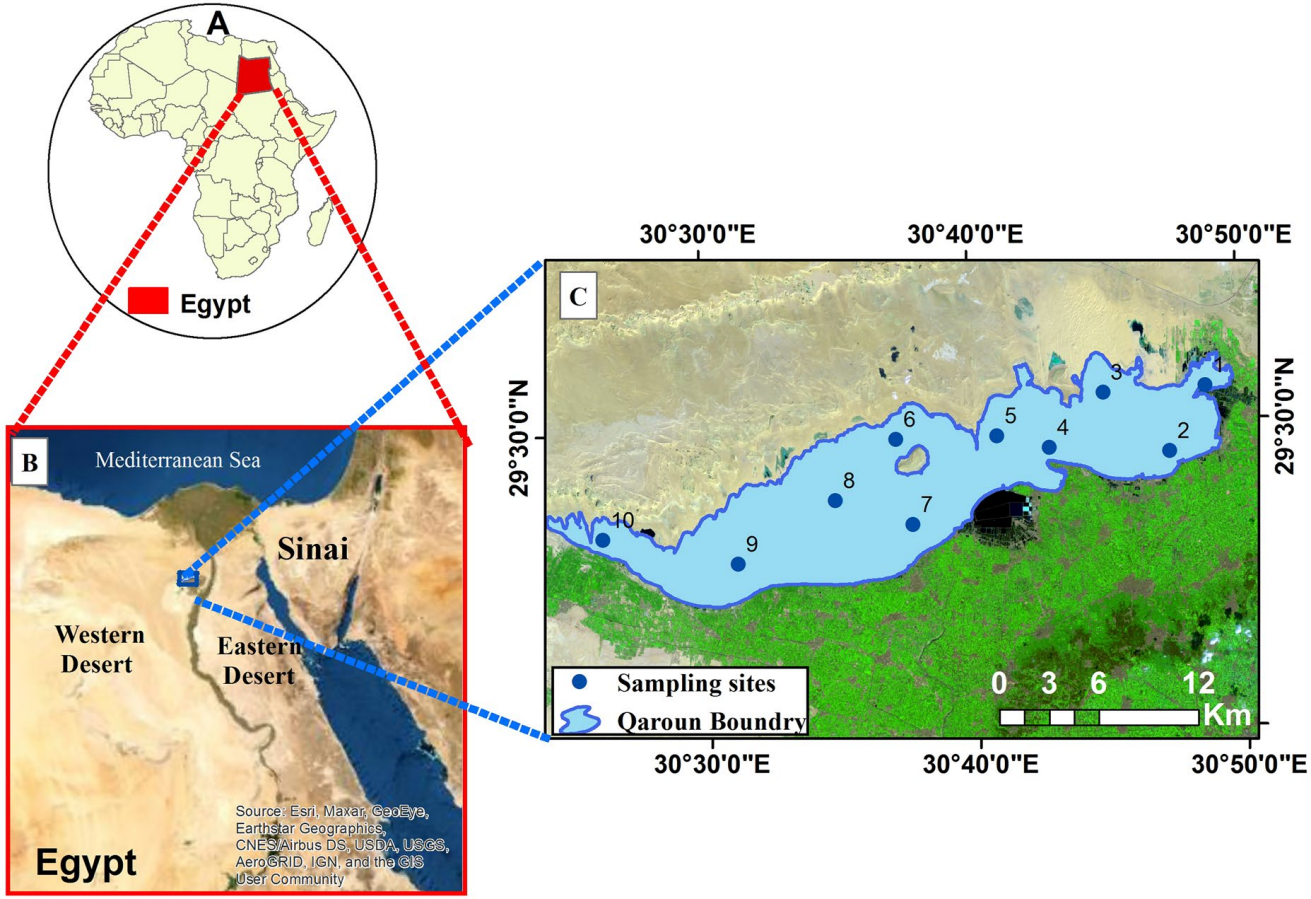
Location map of the study area: **A** African continent where Egypt is located; **B** location map of Egypt showing the boundary of the study area at the north of the western desert; **C** close up view of Lake Qaroun indicating the sampling sites

**Fig. 2:**
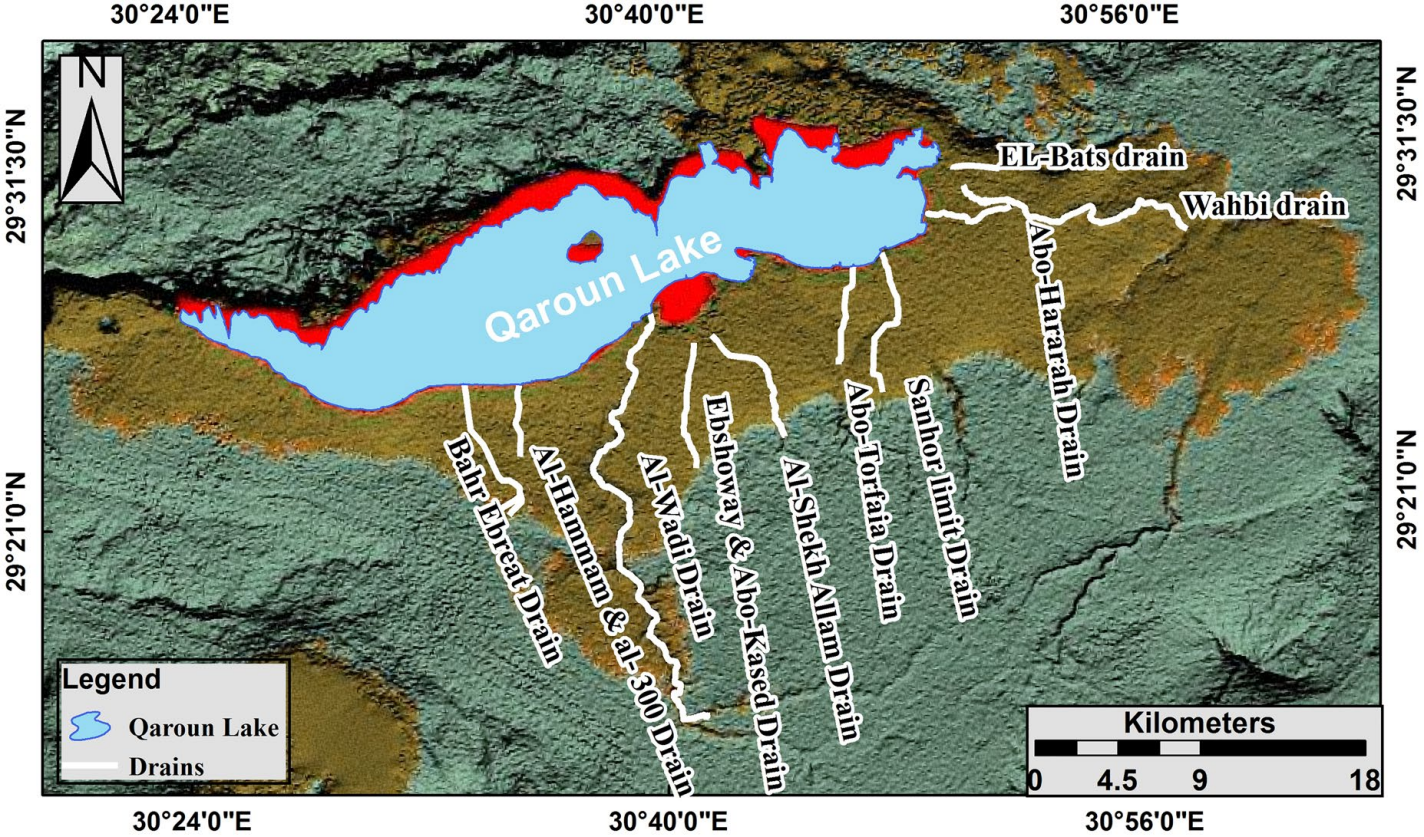
Hill shade of Qaroun Lake and its vicinities illustrating El Fayum depression and the drainage system

Lake Qaroun is located in the North African Sahara Desert. It is a natural lake in the middle of Egypt, definitely in the lowest north-west region of El-Fayoum Depression. It is situated in an arid region between latitudes 30°24′ and 30° 49′ E and latitudes 29° 24′ and 29° 33′ N (Fig. 1). The elevation of Lake Qaroun is located between − 43 and − 45 m below the sea level. Furthermore, the average water depth of the lake is 4.2 m since the Lake is surrounded by residential and agricultural areas from the east and the south, while desert is common at the north. Fresh water enters Lake Qaroun through a drainage network. It has two main drains (El-Bats and Al-Wadi) and a number of secondary drains (Fig. 2). It receives about 450 mm^3^ each year as a mixture of untreated industrial, sewage, agricultural, and domestic effluents from El-Fayum Province (Barakat et al., 2013; El-Zeiny et al., 2019).

### Methodology

**Fig. 3:**
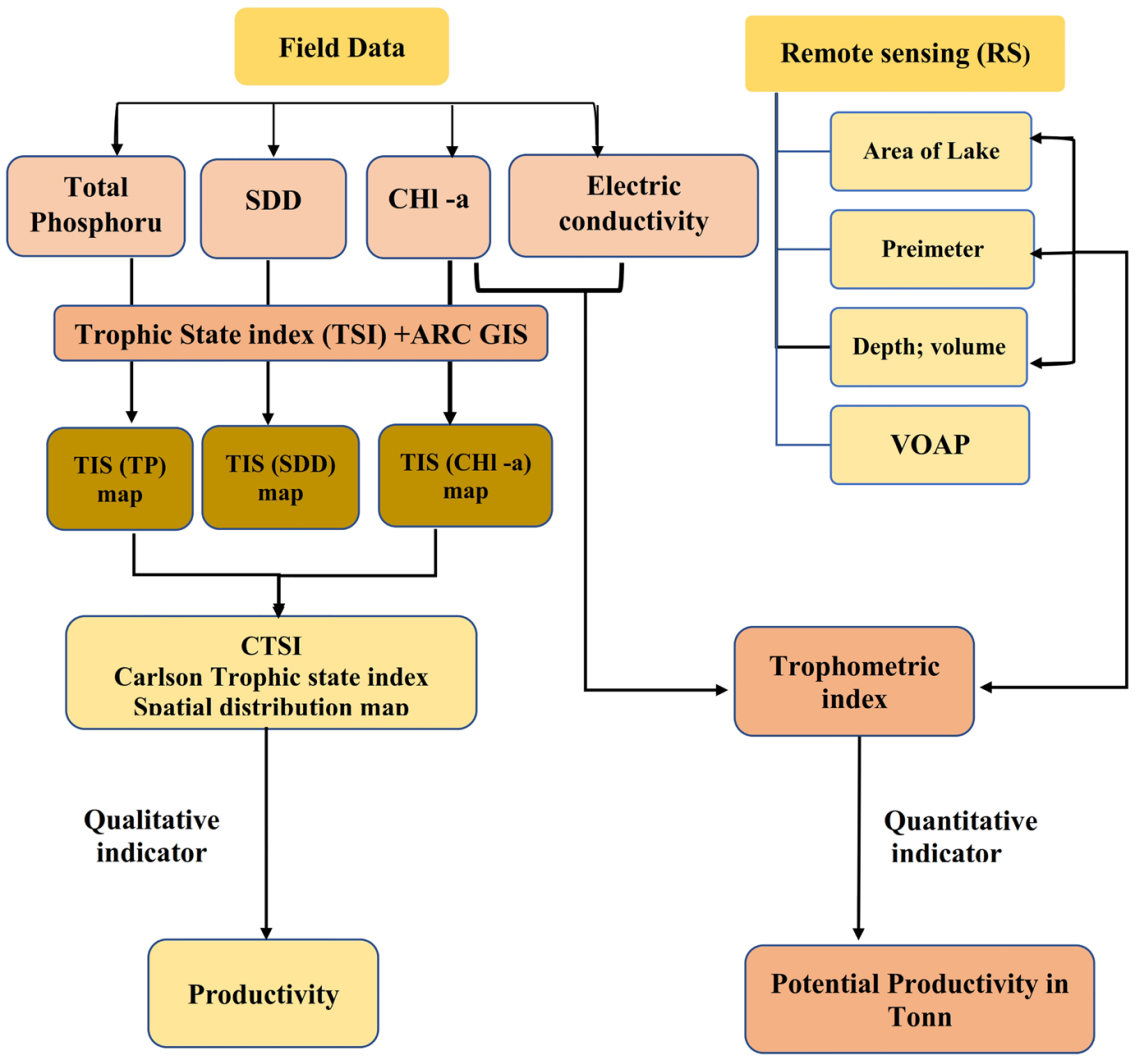
A flowchart showing the methodology adopted in this study

The methodology adopted in the present paper depended on two main inputs: field data and satellite images. Each data set was subjected to several and successive processes, which are summarized in Fig. 3. Each step in the flow chart will be described in detail in the following sections.

### Sampling and analysis

Water samples were collected from ten different sites during November 2018, February 2019, March 2019, and June 2019. The sampling sites are well distributed in Qaroun Lake. All water samples were sent to the laboratory for further analysis (EC and TP) following APHA (1992).

Transparency was measured using a white enameled Secchi disk depth (SDD). Chlorophyll-a determination was performed in the other exact volume of the 500-ml water sample which was filtered on the same day of collection using GF/C filters. The filters were kept in a deep freezer until analysis. Chlorophyll-*a* in the phytoplankton cells retained on the filters was extracted by using 90% acetone and measured spectrophotometrically at 630, 645, 665, and 750 nm wavelengths (Strickland & Parsons, 1972).

The results of the field data were organized in an Excel sheet in order to be imported into ArcGIS 10.5. Some statistical analyses were computed as a minimum, maximum, average, and *Ln* value (natural logarithm) for further use in indices measurements.

#### Index calculation

##### Calculating the TSI

CTSI for lakes is calculated based on some water quality parameters represented in CHL-*a* (μg/L) concentration, total phosphorus TP (μg/L), and water transparency (m). Yet, CTSI ranged from 0 to 100 to assess the trophic state in Lake Qaroun and is classified as driven from (Carlson & Simpson, 1996) (Table 1).

**Table 1:** Category of Carlson trophic state index

**Category**	**Range**
**Oligotrophic**	0–30
**Mesotrophic**	30–50
**Eutrophic**	50–70
**Hyper-eutrophic**	70–100

The following equations were utilized together to describe TSI:

1 $$\mathrm{TSI}(\mathrm{CHL}-\mathrm a)=9.81\;\ln(\mathrm{CH}L-\mathrm a)+30.6$$

2 $$\mathrm{TSI}(\mathrm{SD})=60-14.41\;\ln(\mathrm{SD})$$

3 $$\mathrm{TSI}(\mathrm{TP})=14.42\;\ln(\mathrm{TP})+4.15$$

4 $$\mathrm{CTSI}=\frac{\mathrm{TSI}()+\mathrm{TSI}\left(\mathrm{TP}\right)+\mathrm{TSI}(\mathrm{CHL}-\mathrm a)}3$$

where ln is natural logarithm.

##### Calculation of the PP

The trophometric index was developed by Lara et al. (2009). The trophometric index accommodates seven variables: area (Km^2^), volume (m^3^), average depth (m), VOAP (area/depth) (Eq. 5), EC (mS/cm), CHL-a (μg/L), and perimeter Pe (m) (Eq. 5).

Multiplying inputs into the model helps to increase its accuracy. The inputs of the trophometric index consist of IF (Eq. 5), VOAP (Eq. 6), EC, chlorophyll-a, and VOAP (volume of water to area percent), which is the volume percentage of water with sufficient oxygen to sustain fish life (Wootton, 1990; AKKUŞ & Mustafa, 2019).

5 $$\mathrm{TMI}=\mathrm{IF}\ast\mathrm{VOAP}\ast\ln\;\mathrm{EC}\ast\frac{\ln\;\mathrm{CHL}-\mathrm a}{\ln\;\mathrm{Pe}}$$

where

6 $$\mathrm{IF}=\frac{\mathrm{Area}}{\mathrm{volume}}$$

7 $$\mathrm{VOAP}=\frac{\mathrm{Area }}{\mathrm{depth}}$$

Then, Eq. 8 was used to calculate the potential productivity in kg/ha.

8 $$\mathrm{PP}(\mathrm{Kg}/\mathrm{ha}=-54.191+67.63*\mathrm{TMI}$$

To convert the calculated potential productivity to tons, use.

9 $$\mathrm{PP}(\mathrm{tonnes})=\frac{\mathrm{PP}\left(\mathrm{kg}/\mathrm{ha}\right)*0.405*55340}{1000}$$

### Satellite images and GIS

#### Satellite image acquisition

A high-resolution Sentinel-2B MSI scene was used for carrying out the morphometric analysis, including the lake boundary and perimeter during 2018–2019 for Lake Qaroun (tile number: 35) with pixel size 10 m. The scene was freely downloaded from the Copernicus Open Access Hub website. The database was built from the number of thematic layers that were provided with the UTM Zone 35 projection systems with WGS 84.

#### Geospatial analysis

##### Extraction of Qaroun Lake’s boundary

The normalized difference water index (NDWI) introduced by McFeeters (1996) to detect and map surface water from multi-spectral remote-sensing images (Eq. 10). Calculating the lake’s area could be done through the NDWI, which is a successful index for surface water extraction from the satellite image. Moreover, NDWI has simple, fast, and good achievements through the water indexes that are used for recognizing the water bodies (Jiang et al., 2014). This technique is based on the difference in the absorption and reflection of light between the water and other features in different frequency bands, as described in the following equation.

10 $$\mathrm{NDWI}=\frac{(\mathrm{NIR }-\mathrm{ green})}{(\mathrm{NIR }+\mathrm{ green})}$$

where the green band average range is 0.56 μm. The near-infrared (NIR) reflectance range is 0.84 μm.

The created geodatabase for the extracted lake boundary will be ready to use the information recorded in the attribute table to give area (km^2^) and perimeter (km).

##### Generating thematic maps

The geographic location of each sample was detected using the Global Positioning System (GPS) linked to the measured parameter values in an Excel sheet. The Excel file was subsequently imported into GIS and interpreted using ArcGIS 10.5 in combination with the boundary layer of Lake Qaroun. The kriging interpolation was carried out for the following outputs: TSI **(**SD), TSI (TP), TSI (CHL-a), and CTSI. The concept of Kriging interpolation is to use a limited set of sampled data points to estimate the value of a variable over a continuous spatial field (Oliver & Webster, 1990). In addition, the ordinary kriging approach has been proven that kriging interpolation is the best interpolation method for the water parameters (Huchhe, 2016). Then, using the raster to polygon tool, the classified raster to polygon was used in order to extract the area of each class. Finally, the percentage of each class is calculated through Eq. (11).

11 $$\mathrm{class\%}=\frac{\mathrm{class\;area}}{\mathrm{Total\;Lake\;area}} \times 100$$

## Results

### In situ characterization of water quality

The average water parameter analysis for Lake Qaroun during 2018–2019 is presented in Table 2. Chlorophyll-a levels ranged from 51.9 to 335.8 (µg/L) during the period of the study, with an annual average 156.9 (µg/L). On the other hand, the average annual value of total phosphorus was 102.2 µg/L, with a minimum value of 55 µg/L and a maximum annual value of 295.4 µg/L. The transparency of Qaroun water fluctuated from 0.2 to 0.8 m, and the average value was 0.4 m, while the EC mean value is 45.7 µS/cm, minimum of 30.1 µS/cm, and a maximum of 53.5 µS/cm.

**Table 2:** The average values of water parameters used in this study

**Sample no.**	**TP (µg/L)**	**CHL-*a *(µg/L)**	**SDD (m)**	**EC (µS/cm)**
**1**	295.4	92.7	0.2	30.06
**2**	79.9	116.4	0.4	42.67
**3**	74.7	174.0	0.4	44.0
**4**	82.0	334.2	0.4	48.7
**5**	69.3	335.8	0.5	49.6
**6**	61.2	173.6	0.5	52.5
**7**	186.7	145.7	0.2	30.1
**8**	66.3	77.8	0.7	52.6
**9**	56.9	62.6	0.8	53.0
**10**	55.2	51.9	0.6	53.5
**Average**	102.8	156.5	0.5	45.7
**Minimum**	55.2	51.9	0.2	30.1
**Maximum**	295.4	335.8	0.8	53.5

*TP* total phosphorus, *CHL-a* chlorophyll-a, *SDD* Secchi disk depth, *EC* electrical conductivity

### Morphometric results

Three images of Sentinel-2B MSI scene for February, March, and June were used to extract Qaroun Lake boundary and perimeter through applying the NDWI model. However, data for autumn were taken from Mohamed et al. (2022). The result of the morphometric data required to achieve this work were 231 km^2^ for area and 146.1 m for perimeter.

### Evaluation of CTSI

A Carlson trophic state index was conducted on Qaroun Lake to classify its trophic state. The applied CTSI is represented in Table 3. The overall CTSI is the average values derived from each module: TSI (CHL-*a*), TSI (TP), and TSI (SDD) (Table 3).

**Table 3:**
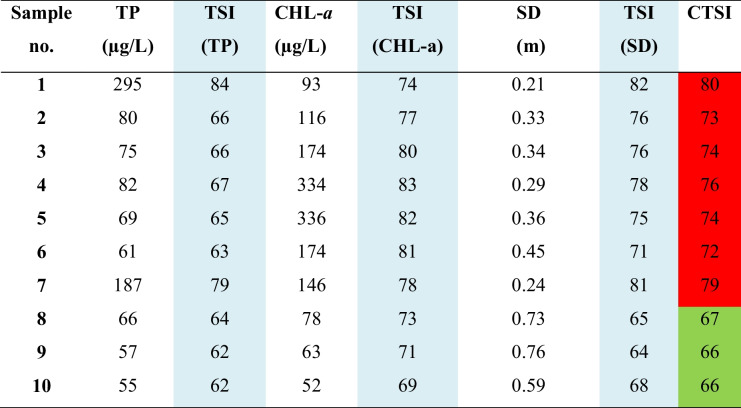
The average TSI (TP), TSI (CHL-a, and TSI (SDD), and CTSI (Carlson & Havens, 2005)

*TP* total phosphorus, *TSI* trophic state Index, *CHL-a* chlorophyll-a, *SDD* Secchi disk depth

**Fig. 4:**
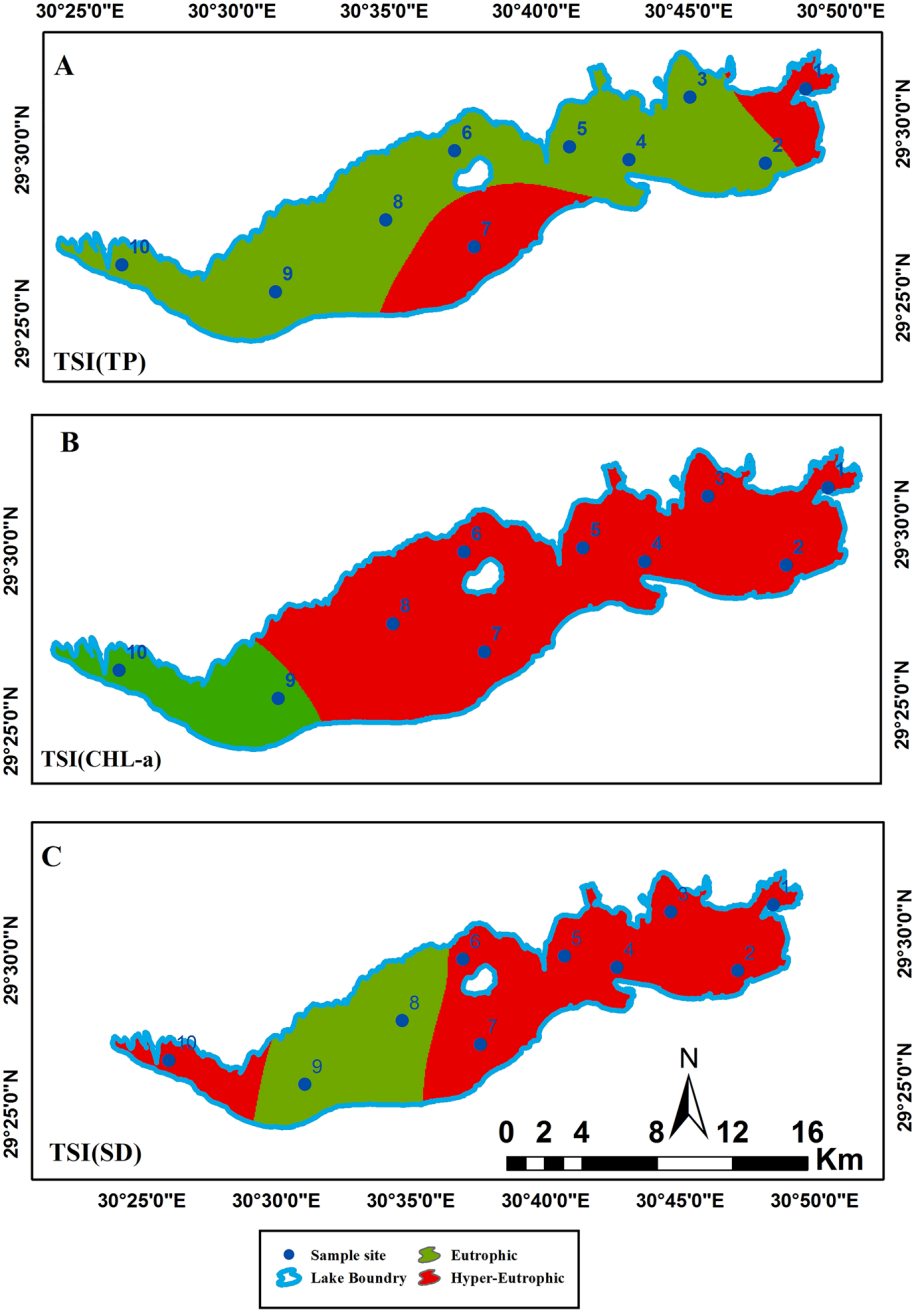
The distribution maps of **A** TSI (TP), **B** TSI (CHL-*a*), and **C** TSI (SD) along Qaroun Lake during 2018–2019

**Fig. 5:**
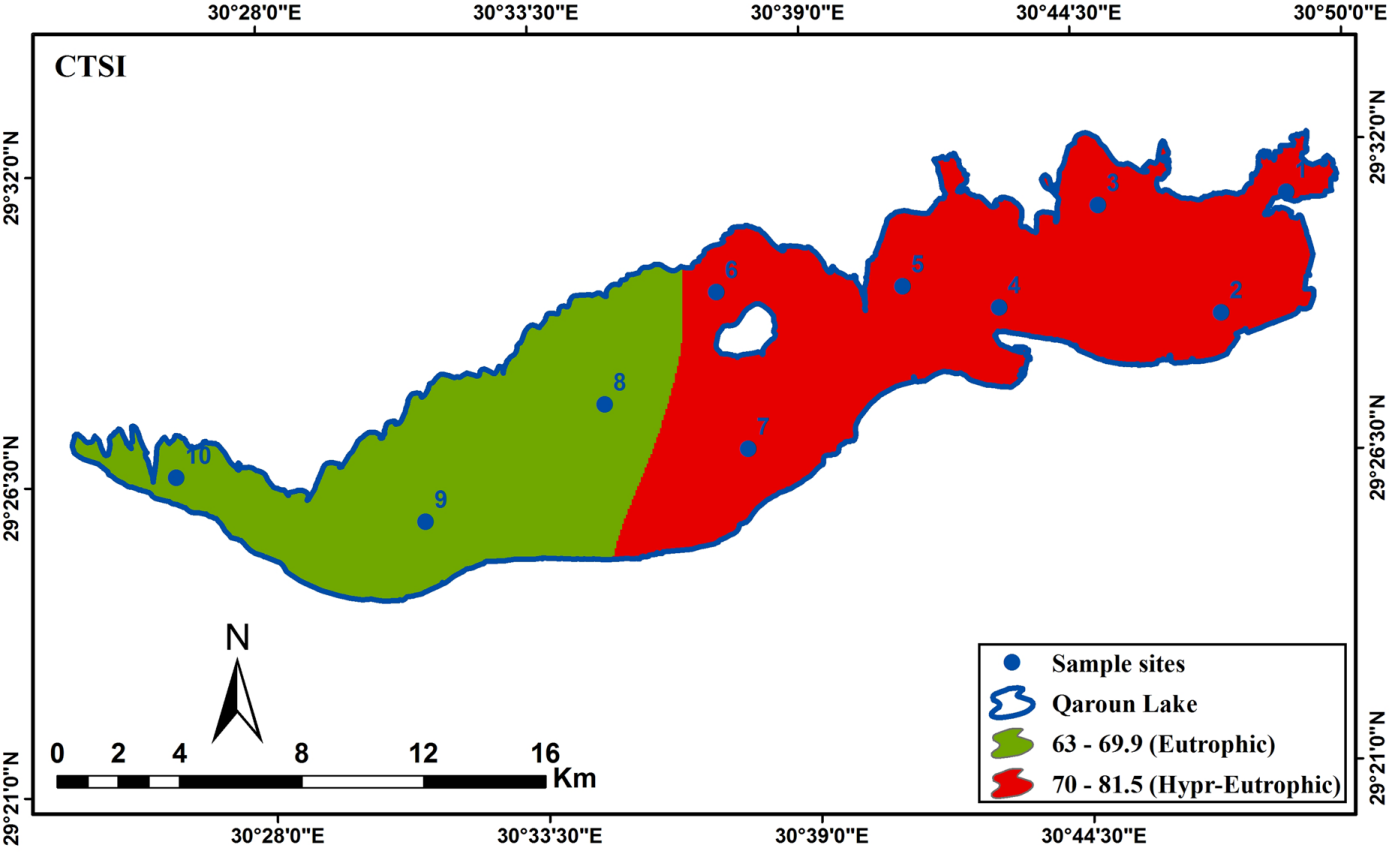
Distribution of overall CTSI along Qaroun Lake during 2018–2019

The spatial distribution maps of the resultant maps are illustrated in Figs. 4 and 5 and Table 4 showing the percentage of each trophic class. The spatial distribution of TSI (TP) showed an obvious hypereutrophic state near the main drains EL-Bats and EL-Wadi drain, representing 21% of the total lake area, while eutrophic state is dominant (79%) as shown in Fig. 4A. The spatial distribution of TSI (CHL-a) showed hypereutrophic areas at the east and the m4iddle of Qaroun Lake, amounting 91% of the total lake area. However, the eutrophic state appeared in the western part of the lake and represents19% of the total lake area (Fig. 4B). TSI (SDD) hypereutrophic state was the dominant class representing 67%. The eutrophic state was 33% around samples 8 and 9 west of the lake (Fig. 4C). The overall CTSI showed the hypereutrophic state with 62% dominating the lake and the eutrophic state with 38% at the west around samples numbers 8, 9, and 10.

**Table 4:**
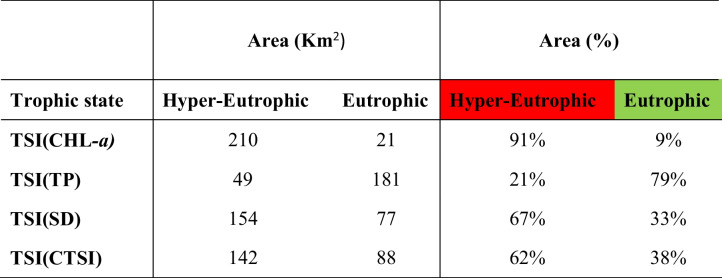
The calculated area of trophic state for each of TSI (CHL-*a*), TSI (TP) and TSI (SDD), and CTSI

*TP* total phosphorus, *TSI* trophic state index, *CHL-a* chlorophyll-a, *SDD* Secchi disk depth

### Potential productivity TMI

The variables required assess and map TMI are described in Table 5. The average potential productivity for the four seasons was estimated using an equation from Lara et al. (2009).

**Table 5:** The values of variable in TMI

Parameters	Nov 2018	Feb 2019	Mar 2019	Jun 2019	Average
Area (km^2^)	228 ^1^	231.2	230.2	230.9	230.8
Perimeter (m)	139	150.5	147.2	147.5	146.1
Volume × 10^8^ (m^3^)	9.804 ^1^	10.034	9.991	10.021	10.0
CHL-a (µg/L)	124.4	101.1	293.6	106.8	156.5
EC (mS/cm)	49.6	36.46	35.29	38.79	40.0
Average depth^1^	4.3	4.3	4.3	4.3	4.3
VOAP	53%	53.70%	53.50%	57.70%	54%

*CHL-a* chlorophyll-a, *EC* electrical conductivity

(1) Mohamed et al. (2022). VOAP (volume of water to sustain life in area percent) was estimated by multiplying the total area of each reservoir by the depth at which the oxygen is equal to 3.0 mg L)^1^, considered by Wootton (1990) as the tolerance limit for the survival of fish

$$\mathrm{IF}=\frac{230.8}{10}$$

$$\mathrm{TMI}=0.24 \times 54\mathrm{\% }\times 3.68 \times \frac{5.05}{4.98};$$

$$\mathrm{PP}(\mathrm{kg}/\mathrm{ha})\:=\:54.191\:+\:67.63\:\times\:1.3;$$

$$\mathrm{PP}(\mathrm{tons})=\frac{34.4\;\times\;0.405\;\times\;\mathrm{56,340}}{1000}$$

$$\mathrm{PP}(\mathrm{tons})=652\mathrm t.$$

## Discussion

### Trophic state of Qaroun Lake

Eutrophic is a common phenomenon in inland lakes (He et al., 2021). Qaroun Lake’s trophic state has been previously discussed in many ecological studies as a eutrophic lake or highly eutrophic state. They attributed the eutrophic state of the lake to receiving excess nutrients, which increases the chlorophyll-a concentration rates. Additionally, Qaroun Lake is located within an arid to hyperarid region that is characterized by high temperatures and thus high evaporation rates with stable water movement (Abu-Ghamja et al., 2018; Goher et al., 2018; Ibrahim et al., 2021). On the other hand, a trophic level index (TLI) of Qaroun Lake was carried out for specific sites by Napiórkowska-Krzebietke et al. (2016) which revealed that the status of the lake is a hypereutrophic state in most of the sampling sites. However, in the current study, the calculated trophic state of the lake was missing the oligotrophic and mesotrophic states. It ranged between eutrophic and hyper-eutrophic state, occupying 38% and 62% of the surface area of the whole lake respectively. Based on the calculated trophic state for each sampling site in the study area, a clear 2D map for the spatial distribution of the TSI (TP), TSI (CHL-*a*), TSI (SDD), and CTSI has been created using the potential of GIS. Investigation of the TSI (TP) map shows that the zones of hyper-eutrophic state appear closer to the two discharge points represented by the two main drains that are connected to Qaroun Lake (i.e., El Bats; El Wadi). This is due to the fact that phosphorus enters the natural water resources from external sources, particularly agricultural drainage wastes (Mahmoud, 2015). The agriculture drains in Egypt are loaded with excessive amount of pesticides, which carry a high amount of TP (Matter et al., 1992). Phosphorus is an essential element for accelerating the phytoplankton growth (Saputra et al., 2017). Exceeding the concentration of phosphorus will seriously increase the growth of phytoplankton, leading to the appearance of a hyper-eutrophic state (Saetang & Jakmunee, 2021).

Consequently, the examination of the generated spatial distribution map of TSI (CHL-*a*) shows that most of the surface area of the lake falls within the hyper-eutrophic state covering 91% from the total area of the lake. The chlorophyll levels ranged between 51.9 and 335.8 µg/L which exceeded the standard chlorophyll-a levels for the lake’s water, where the normal levels are ranging between 4.49 and 69.50 μg/L (Saeed & Mohammed, 2012). It is worthy mentioned that the chlorophyll levels are proportionally increasing during the last 8 years where in 2008, the average chlorophyll-a concentration was 201.5 µg/L, while in March 2015, the recorded CHL-a average reaches its maximum value 336 μg/L showing a blooming phenomenon (Hussein et al., 2008; Zaher & Ibrahim, 2018). High CHL-a concentrations give alarm to increase lake eutrophication where chlorophyll-*a* is positively correlated to enriched nutrient concentrations which forms a blooming, poor water quality and the presence of harmful species of algal. Carlson (1977) confirmed that the best indicator in assessing trophy is chlorophyll a.

Finally, transparency will be affected due to the vigorous growth of phytoplankton. Using TSI (SDD), the hyper-eutrophic state was 67%. The transparency factor in Qaroun Lake is low due to the direct discharges of two main drains carrying the suspended particles (e.g., fertilizers and, fish farming outputs, the disposal of untreated sewage, and agricultural effluents) to the lake. The combination of the three modules of trophic states (TP, CHL-a, and SSD) derived the CTSI map showing the overall eutrophic state as 38% and the hyper-eutrophic state as 62%. The negative impacts of hyper-eutrophic are arisen from the decline in oxygen level in shallow lakes which create dead zones that change the ecosystem, decrease biodiversity, and have a dramatic effect on the fish productivity of the water body (Fathi & Flower, 2005; Khalil et al., 2017; El-Zeiny et al., 2019). On the other hand, in a nearby lake in the study area (Wadi El Rayan Lakes), only the TSI (CHL-a) from the parameters of CTSI was applied to detect the trophic sate for the two lakes. The study revealed that the first lake is mesotrophic, while the second Wadi El Rayan lake is oligotrophic according to Konsowa (2007) which presented normal levels of trophic state that may be attributed to the absence of draining system reaching water bodies, keeping them in normal conditions.

#### Estimation of the potential productivity using TMI

In the current study, the average annual potential productivity (PP) based on trophometric index (TMI) was 652 t during 2018–2019. Indeed, the advantage of the potential productivity model is that it could clarify the gap between the actual yield (832 t) reported from CAPMAS (2018) and the estimated PP (652 t). The accuracy of the applied model for estimating potential productivity is 80% of the actual yield indicating an acceptable level of accuracy. The advantage of the TMI is the contribution of seven vital variables to identify the productivity giving more reliable results and estimations.

In closure, it can be mentioned that the low actual productivity of the lake is greatly affected by the detected hyper-eutrophication state resulting from the environmental related impacts of untreated discharges (sewage and agriculture). Moreover, it is necessary to restore this closed basin to maintain a suitable environment for the living organisms and work on the sewage treatment before discharging into the lake.

## Conclusion

The present study achieved the multidisciplinary integration of in situ measurements, environmental indices, and GIS to assess the spatial distribution of the lake’s trophic state. Moreover, it focused on applying models such as CTSI to assess the trophic state of the lake and the TMI to assess potential productivity. According to CTSI, TP was the main factor for the trophic state of the lake. The degree of annual and potential productivity from the trophometric index (TMI) indicated an acceptable accuracy for estimating Qaroun Lake yield that can widely be used in sustainable management plans. On the other hand, it is necessity to work on raising the lake potentiality and decreasing the level of pollution reaching the lake particularly nutrients levels.

Accordingly, it is highly recommended to pay more attention to Lake Qaroun as a closed system exposed to excessive pollution that threatens productivity, i.e., loss of species diversity and quantitatively changes (i.e., decrease in fish production). Providing triple wastewater treatment plants is a must to improve drains water quality and consequently restore Lake Qaroun.

The present study provides the decision maker with the necessary thematic layers and spatial analyses that is necessary for future planning and productivity improvement. The current multidisciplinary integration achieved satisfactory results and is applicable in similar settings.

## Data Availability

All data generated or analyzed during this study are included and available in this article.
